# Maternal ancestry and lineages diversity of the Santander population from Colombia

**DOI:** 10.1093/fsr/owad032

**Published:** 2023-09-21

**Authors:** Adriana Castillo, Fernando Rondón, Gerardo Mantilla, Leonor Gusmão, Filipa Simão

**Affiliations:** Department of Basic Sciences, Genetics Laboratory, Industrial University of Santander, 680002, Bucaramanga, Colombia; Department of Basic Sciences, Genetics Laboratory, Industrial University of Santander, 680002, Bucaramanga, Colombia; Department of Basic Sciences, Genetics Laboratory, Industrial University of Santander, 680002, Bucaramanga, Colombia; DNA Diagnostic Laboratory, Institute of Biology Roberto Alcantara Gomes, State University of Rio de Janeiro, 20550-900, Rio de Janeiro, Brazil; DNA Diagnostic Laboratory, Institute of Biology Roberto Alcantara Gomes, State University of Rio de Janeiro, 20550-900, Rio de Janeiro, Brazil

**Keywords:** forensic genetics, mitochondrial DNA, native ancestry, substructure

## Abstract

Santander, located in the Andean region of Colombia, is one of the 32 departments of the country. Its population was shaped by intercontinental admixture between autochthonous native Americans, European settlers, and African slaves. To establish forensic databases of haplotype frequencies, the evaluation of population substructure is crucial to capture the genetic diversity in admixed populations. Total control region mitochondrial deoxyribonucleic acid haplotypes were determined for 204 individuals born in the seven provinces across the department. The maternal native heritage is highly preserved in Santander genetic background, with 90% of the haplotypes belonging to haplogroups inside A2, B4, C1, and D. Most native lineages are found broadly across the American continent, while some sub-branches are concentrated in Central America and north South America. Subtle European (6%) and African (4%) input was detected. In pairwise comparisons between provinces, relatively high *F*_ST_ values were found in some cases, although not statistically significant. Nonetheless, when provinces were grouped according to the principal component analysis results, significant differences were detected between groups. The database on mitochondrial deoxyribonucleic acid control region haplotype frequencies established here can be further used for populational and forensic purposes.

## Introduction

**Figure 1:**
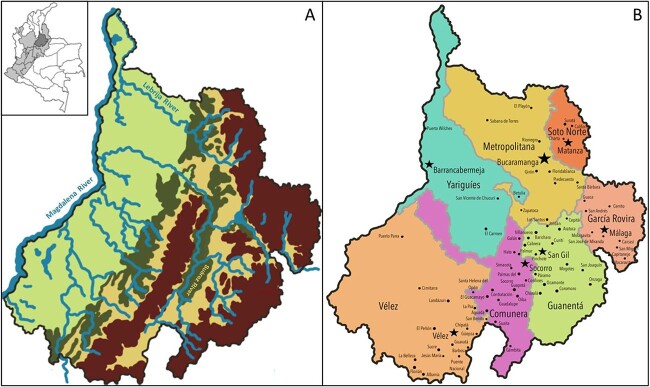
(A) Relief map of Department of Santander depicting the mountainous highlands of Eastern Andes range (brown), rivers (blue), and flat lowlands (different shades of green). The map on the upper left corner illustrates Colombia, with focus on the Andean region (lighter grey) and Santander department (darker grey). (B) Department of Santander with provinces highlighted at different colours. The municipalities are represented by black dots and the capital of each province is represented by black stars.

Santander is a Colombian department, located in the Andean region, in the northeast of the country (Figure 1A). In pre-Columbian times, the territory was occupied by natives belonging to several ethnolinguistic groups, mainly from the Chibcha family. The Guanes, who were the most numerous, lived in the middle downstream part of the Suárez River (Figure 1A). Also inhabiting the region were the Chitareros and the Lache that occupied the highlands of the Eastern mountains, and the Yareguies that occupied a valley region of the Magdalena River (Figure 1A).

During the European conquest (which began in the 16th century), the Spanish invaders arrived in the territory of Santander, entering from the north through the banks of Magdalena and Lebrija rivers (Figure 1A). As they advanced through the rivers, two types of settlements took place. Upon their arrival, the European settlers occupied territories already inhabited by natives, to carry out their evangelization. In these settlements, admixture took place essentially between European men and native women. New settlements were also established in unoccupied areas, initially forming communities of Europeans. These new communities of Creoles (Spanish descendant born on Colombia) gradually incorporated other people of native American, African, and admixed heritage, eventually becoming admixed populations. These two types of settlements grew and gave rise to the different municipalities that exist in Santander today [1].

From the 16th to 19th centuries, during the transatlantic slave trade, millions of Africans arrived at the Colombian coasts of Caribe and, to a lesser extent, of Pacific (https://www.slavevoyages.org/). While most of the African slaves were taken to coastal territories to work on mining and on sugar cane and coffee plantations, few were also brought to Santander, through the Magdalena River [2]. Over time, admixture processes took place between the different ethnicities occupying the territory [3, 4]. The patterns of admixture occurred differently across the country. This resulted in a high genetic differentiation between and within the different Colombian departments [5, 6].

Santander was officially founded in 1 857 and currently comprises 87 municipalities distributed in seven provinces: Comunera, García Rovira, Guanentá, Metropolitana (which includes the capital Bucaramanga), Soto Norte, Vélez, and Yariguíes (Figure 1B) [7]. The department is crossed from south to north by the Eastern branch of the Colombian Andes), and the population is concentrated in the urban areas, over the mountainous region. The topography is rough and uneven, formed by areas of highland combined with areas of flat lowlands, which difficult the access to some provinces.

In Colombia, the need for human identification in armed conflict incidents is common. In 2016, a peace agreement was signed between the Colombian government and the *guerrillas* (small organized armed groups that proclaim the defence of the rights of the people, acting outside the law). This resolution included a commitment of identification by the state of the disappeared victims from armed conflicts [8].

Most forensic caseworks are routinely solved using autosomal Short Tandem Repeats (STR) markers. However, additional (nonautosomal) markers can be required in complex kinship investigations (e.g. with no direct relatives), or when degraded and/or low template DNA samples are available for forensic identification (e.g. biological stains, bone remains, or hair). In these situations, mtDNA (mitochondrial DNA) has become an important tool. The lack of recombination and high copy number make mtDNA useful in determining distant maternal kinships, as well as in obtaining genetic profiles from degraded samples [9]. As for other genetic markers, the implementation of mtDNA haplotype frequency databases is required to accurately weight evidence. The accuracy of these databases depends on both sample size as well as on the representativeness of the full genetic composition, considering possible population substructure. Accounting for population substructure is even more important in admixed populations that resulted from mating among individuals from different continents and/or ethnolinguistic groups, as is the case of most South American populations.

Except for a preliminary study on the Andean region of Colombia [10], there are, currently, no mtDNA control region (CR) databases for Colombian admixed populations (including Santander). Published data are only available for Restriction Fragment Length Polymorphisms (RFLPs) or some hypervariable segments of the CR (HVSI and/or HVSII) [11–13]. Data on the full CR are only available for native American or Afro-descendant populations [14–17].

Therefore, the aim of this study was to determine the maternal lineages’ composition of Santander population, by analysing mtDNA CR in samples from all provinces of the department. Results were used to investigate interpopulation differentiation and to estimate forensically relevant parameters. The data obtained were submitted to the European DNA Profiling Group mtDNA Population Database (EMPOP) [18] for quality control and are available for investigation purposes in forensic and population genetic fields.

## Material and methods

### Population sample

Blood samples were collected from 204 unrelated individuals born in Santander department (Colombia). From those, 45 samples were included in a preliminary study presented at a congress [10].

Samples were selected to represent the seven provinces of Santander: Comunera (*n* = 30), García Rovira (*n* = 30), Guanentá (*n* = 28), Metropolitana (*n* = 36), Soto Norte (*n* = 26), Vélez (*n* = 23), and Yariguíes (*n* = 31). Considering the distribution of the urban areas and their population densities, most samples were from West Santander (Figure 1B).

### Mitochondrial DNA typing

DNA was extracted using PureLink™ Genomic DNA Mini Kit (ThermoFisher Scientific). Mitochondrial DNA CR, located between positions 16024 and 576, was amplified in a single amplicon, using the primers L15900 (5′-CACCATTAGCACCCAAAGCT-3′) and H639 (5′-GGGTGATGTGAGCCCGTCTA-3′). PCR was performed in a final volume of 5 μL, with 1–5 ng of DNA, 1X master mix from the Qiagen® Multiplex PCR kit (Qiagen), and 0.2 μmol of each primer. The thermocycling conditions were initial denaturation at 95°C for 15 min; 35 cycles at 94°C for 30 s, 60°C for 90 s, and 72°C for 60 s; and a final extension at 72°C for 10 min. The amplified product was purified with ExoSAP-IT (Applied Biosystems). Sequencing was performed using the BigDye Terminator v3.1 cycle Sequencing kit (Applied Biosystems), according to manufacturer guidelines. The primers used were L15900, L16555 (5′-CCCACACGTTCCCCTTAAAT-3′), H159 (5′-AAATAATAGGATGAGGCAGGAATC-3′), and H639. The thermocycling protocol was: denaturation at 96°C for 2 min; followed by 35 cycles at 96°C for 15 s, 50°C for 9 s, and 60°C for 2 min; and a final extension at 60°C for 10 min. When double coverage was not achieved due to length heteroplasmy, additional sequencing was performed using one or more of the following primers: L16268 (5′-CACTAGGATACCAACAAACC-3′), H016 (5′-CCCGTGAGTGGTTAATAGGGT-3′), H599 (5′-TTGAGGAGGTAAGCTACATA-3′), L314 (5′-CCGCTTCTGGCCACAGCACT-3′), R338 (5′-GTTTAAGTGCTGTGGCCAGAAG-3′), and R484 (5′-TGAGATTAGTAGTATGGGAG-3′). A final purification of the sequenced products was performed using Sephadex® G-50 (Sigma-Aldrich). Separation and detection were achieved by capillary electrophoresis on an ABI 3500 (Applied Biosystems).

### Haplogroup assignment

Haplotypes were classified using the software SeqScape v2.7, by comparison with the revised Cambridge Reference Sequence [19]. Haplotypes were further revised with the EMPOP Alignment tool [18]. Haplogroups were assigned on EMPOP according to Phylotree build 17, February 2016 [20]. The quality of the mtDNA dataset was evaluated using the “EMPcheck” tool available on EMPOP [18]. The dataset was submitted to EMPOP for internal quality control [18] and is available for forensic searches under the accession number EMP00862.

**Table 1:** Haplogroup frequencies found in Santander department, Colombia.

Haplogroup	*n*	%
A2	4	1.96
A2+(64)	35	17.16
A2+(64)+@153	3	1.47
A2+(64)+16129	3	1.47
A2ac	21	10.29
A2af1a1	2	0.98
A2r1	2	0.98
A2v	5	2.45
A2y	1	0.49
B2b3a	2	0.98
B2d	60	29.41
B2h	1	0.49
B4b	1	0.49
B4	1	0.49
C1	10	4.90
C1b	1	0.49
C1c5	4	1.96
C1d+194	3	1.47
D	1	0.49
D1	8	3.92
D1a2	2	0.98
D1f	10	4.90
D4h3a	4	1.96
**Total Native**	**184**	**90.20**
J1c	3	1.47
K1a	1	0.49
R0	3	1.47
U5a2	1	0.49
U5b	1	0.49
**Total European**	**12**	**5.88**
L0a1a+200	1	0.49
L1b	1	0.49
L1c1'2'4'5'6	1	0.49
L2a1	1	0.49
L2a1+143	1	0.49
L3b	1	0.49
L3d5a	1	0.49
L3f1b1a1	1	0.49
**Total African**	**8**	**3.92**

### Statistical analysis

Haplogroup frequencies were calculated by direct counting. Haplotypes were converted into aligned sequences using HaploSearch [21]. The Arlequin software [22] was used to calculate haplotype diversity (H), mean number of pairwise differences (MNPD), nucleotide diversity (π) and mismatch distribution, as well as to perform population comparisons (by mean of *F*_ST_) and analysis of molecular variance (AMOVA). The exclusion power (mtCE) was calculated according to Simão et al. [23]. The software STATISTICA (data analysis software system) 10.0.0.15 (TIBCO Software Inc., Palo Alto, CA, USA) was used in the multidimensional scaling representation of pairwise genetic distances and to perform principal component analysis (PCA) based on haplogroup frequencies. Phylogenetic networks were designed with median-joining method [24] on Network 10.0 software (Fluxos Technology Ltd, Colchester, UK), using mtDNA haplotypes without considering indels at positions 16193, 309, 315, 523–524, and 573. These indels are associated to homopolymeric tracts and are therefore commonly ignored in forensic comparisons and database searches [25].

## Results and discussion

The mtDNA CR haplotypes and corresponding haplogroups are detailed in Supplementary Table S1. Most samples have native American maternal ancestry (90%) classified inside macrohaplogroups A2, B4, C1, and D. The percentage of lineages of European and African origin was low, with just 6% belonging to branches inside macrohaplogroup R and 4% inside L0–L3 (Table 1).

Point heteroplasmy was detected in 10 samples (5%), at nine different positions, as described in SupplementaryTable S2. Except for a C/T at position 16211, the point heteroplasmies detected in this study were previously reported in other populations, available on the EMPOP database (v4/R13, accessed 25 May 2022). Length heteroplasmy was detected in 45% of the haplotypes. In 27 samples, length heteroplasmy was detected in more than one site. Most of the occurrences were on poli-C tracts at HVSI and HVSII. In HVSI, most length heteroplasmies resulted from a T/C substitution at 16189 together with C-insertions at position 16193 and/or A/C substitutions at positions 16182–16183. In HVSII, length heteroplasmy was associated with C-insertions at 309 and/or 315. Although less frequent, length heteroplasmies were also detected due to C-insertions at 463 and 573 positions (2%), and in AC repeats between positions 515–524 (2%).

Rare indels were detected at positions 89 (samples S034 and S133), 106 (sample S010), 116 (samples S096 and S104), and 489 (sample S001).

### Population diversity

A total of 148 different CR haplotypes were detected, 125 of which were unique. The haplotype diversity was 0.9925 ± 0.0020 and the exclusion power was 0.9673. The MNPD between haplotypes was 15.1850 ± 6.8146, resulting in a nucleotide diversity (π) of 0.0133 ± 0.0066 (Table 2). These values are in the range of those previously reported for other South American admixed populations (Supplementary Table S3), including those from the neighbour countries of Venezuela and Ecuador (Supplementary Table S3).

**Table 2:** Diversity parameters calculated for Santander department, Colombia.

Item	*n*	H	Singletons (%)	mtCE	MNPD	*π*
All positions						
All	204	0.9925 ± 0.0020	125 (61)	0.9673	15.1850 ± 6.8146	0.0133 ± 0.0066
Comunera	30	0.9885 ± 0.0126	23 (77)	0.9379	14.7471 ± 6.7895	0.0131 ± 0.0067
García Rovira	30	0.9885 ± 0.0126	23 (77)	0.9517	14.3080 ± 6.5966	0.0127 ± 0.0065
Guanentá	28	0.9947 ± 0.0112	24 (86)	0.9868	14.9339 ± 6.8859	0.0132 ± 0.0068
Metropolitana	36	0.9921 ± 0.0084	26 (72)	0.9746	15.7270 ± 7.1849	0.0139 ± 0.0071
Soto Norte	26	0.9846 ± 0.0160	19 (73)	0.9815	16.4585 ± 7.5752	0.0146 ± 0.0075
Vélez	23	0.9881 ± 0.0186	20 (87)	0.9644	14.3874 ± 6.6912	0.0128 ± 0.0066
Yariguíes	31	0.9914 ± 0.0116	26 (84)	0.9462	14.4989 ± 6.6742	0.0128 ± 0.0066
Excluding indels						
All	204	0.9783 ± 0.0055	95 (47)	0.9293	13.1026 ± 5.9220	0.0116 ± 0.0058
Comunera	30	0.9724 ± 0.0209	21 (70)	0.9034	12.6851 ± 5.8837	0.0113 ± 0.0058
García Rovira	30	0.9586 ± 0.0269	21 (70)	0.9149	12.5678 ± 5.8321	0.0112 ± 0.0058
Guanentá	28	0.9868 ± 0.0141	21 (75)	0.9127	12.7169 ± 5.9100	0.0113 ± 0.0059
Metropolitana	36	0.9714 ± 0.0190	24 (67)	0.9476	13.8556 ± 6.3670	0.0123 ± 0.0063
Soto Norte	26	0.9846 ± 0.0160	19 (73)	0.9723	14.3200 ± 6.6318	0.0128 ± 0.0066
Vélez	23	0.9802 ± 0.0198	16 (70)	0.9447	12.3794 ± 5.8011	0.0110 ± 0.0058
Yariguíes	31	0.9548 ± 0.0311	24 (77)	0.8903	12.3183 ± 5.7171	0.0110 ± 0.0057

H: haplotype diversity; mtCE: exclusion power; MNPD: mean number of pairwise differences; *π*: nucleotide diversity.

Mismatch profiles displayed multimodal distributions in all populations (Supplementary Figure S1). These multimodal distributions showed haplotypes with a low number of differences (0–8) together with haplotypes with >10 differences. This profile is common in stabilized populations, contrarily to unimodal distributions that are characteristic of expanding populations [26, 27]. Recent immigration movements of a considerable number of individuals with a different genetic background can also explain multimodal mismatch distributions. Considering the history and recent demography of the Santander population, both phenomena must be behind the observed profiles.

Part of the observed diversity was due to indel polymorphisms located at homopolymeric tracts. Because some forensic laboratories chose to discard these tracts [25], the diversity parameters were also calculated after excluding these polymorphisms. The diversity values decreased considerably (Table 2). Among the 204 samples analysed 124 different haplotypes were found, of which 95 were only detected once.

When not stated otherwise, the following analyses were performed using the CR haplotypes without considering indels at positions 16193, 309, 315, 523–524, and 573.

### Maternal lineages in Santander

The input of non-native maternal lineages in Santander was low, corresponding to only 10% of the haplotypes. The African ancestry (4%) was represented by eight haplotypes classified inside eight different L-branches (Table 1). To trace the origin in Africa of the L-lineages found in Santander, shared haplotypes were searched among samples from Santander and several regions of Africa [28–36]. Only three haplotypes showed matches within Africa. L1b and L3f1b1a1 haplotypes matched samples from Central-West and South-Central Africa while the L3b haplotype showed a wide distribution, from West to East Africa. Because of the poor representativeness of African haplotypes in databases, tracing the origin of the L-lineages to specific African regions is a challenging task. Previous studies [15, 35] pointed to the need of more studies on the maternal background of African populations that accurately represent the high diversity existing in the continent.

The 12 haplotypes detected with European ancestry (6% of the samples) were less diverse than the African. Identical sequences were found inside branches H, J1c, and R0. All European haplogroups detected can be found at high frequencies across Europe. Taking into consideration the historic settlement of Colombia, most of these haplotypes came most probably from the Iberian Peninsula. In fact, a search on EMPOP showed that the J1c and U5b haplotypes found in Santander were only detected in samples from Portugal and Spain. Interestingly, among the 5 623 haplotypes from haplogroup H deposited on EMPOP, only three matches were found with the Santander samples, all reported in the American continent.

The higher African than European haplotype diversity can be related to the diversity of the populations of origin (African populations are the most diverse in the world, and African people were taken to America from different source populations). Other explanation could be the higher number of African than European females that arrived in Colombia during the colonial period.

Native American lineages comprised most of the ancestry in Santander department (Table 1). Similar values of maternal native ancestry were previously reported for Santander (91.0%) [6]; while a lower proportion was found by Yunis and Yunis [11] (82.6%) (Supplementary Table S4). This discrepancy can be due to different sampling strategies.

Since the results available on maternal ancestry of Colombian populations (admixed, Afrodescent, and native) rely mostly on RFLPs, a thorough comparison with this study was hampered. Nonetheless, data on macrohaplogroup frequencies (Supplementary Table S4) show variation on the native ancestry proportion among departments pointing to different admixture patterns across the country. By looking at Supplementary Table S4, a high variation on macrohaplogroup frequencies is also clear among Colombian populations from different geographic regions or ethnolinguistic backgrounds.

Similar proportions of native mtDNA haplogroups (>80%) were reported in western Andean populations from South America and in Paraguay. The native ancestry decreases in central and eastern populations, reaching the lowest values in populations in the Atlantic coast of Brazil (<35%) [23, 37–39].

### Native American haplogroups in Santander

Branches inside haplogroups A2 and B4 were the most common in Santander, in particular the lineages B2d, A2+(64), and A2ac, which together account for 63% of the native haplogroups found in the present study.

The B2d, characterized by a deletion in position 498, was found in 60 samples. A low diversity of sequences was detected, with an MNPD of 1.35. The network representation of this branch (Supplementary Figure S2) showed that sequences differ from each other by a maximum of five positions. A high number of shared sequences could be observed, which corroborates the low intra-haplogroup variability. The star-like distribution of this branch, together with the low variability detected, point to bottleneck and/or isolation events. This lineage is more frequent in populations from lower Central America and from the northern part of South America (unpublish data available on EMPOP database, v4/R13, accessed 25 May 2022), indicating some degree of geographic specificity.

According to the Phylotree ([20]; Build 17, 18 February 2016), A2+(64) haplotypes share the T146C, C152T, A153G, and C16111T polymorphisms with A2 haplotypes, and have an extra one at position 64. Inside this haplogroup, two of the haplotypes from Santander did not include the 153G and other four lacked the transition at 16111. The network distribution of this branch (Supplementary Figure S3) showed a high variety of haplotypes that differed from each other by 11 positions in average. The A2+(64) haplogroup was previously described in native and admixed populations from all over America, without a geographic or linguistic specificity [16, 29, 40–42].

The haplotypes inside A2+(64) that exhibit a G/A transition in position 16213 were classified as A2ac. Fourteen out of the 21 A2ac samples from Santander had the same CR sequence (after discarding indels at homopolimeric tracts) (Supplementary Figure S4). Most of the A2ac samples on EMPOP (v4/R13, accessed 25 May 2022) belong to the Colombian population from Antioquia. Few were also detected in Ecuador and in the USA. Like B2d branch, A2ac also displayed a star-like distribution (Supplementary Figure S4).

The lineage D1f was found at a lower frequency than those previously detailed. This lineage seems to be specific of northern South American populations, with the highest frequencies reported in Ecuador, Colombia, Perú, and Venezuela [14, 16, 43–46]. Among the 10 samples detected within this haplogroup, six shared the same haplotype.

The native subhaplogroups found in Santander show distinct representation in different ethnic groups. Some branches (e.g. A2) are scattered across populations from different geographic locations and ethnolinguistic groups [16, 47, 48]. Other subhaplogroups (e.g. A2+64+154 and A2ac) are restricted to Colombia and surrounding countries [16, 45]. Some lineages reported in Santander (e.g. B2b3a) are typical of native groups from Eastern South America [47, 48]. Finally, other lineages, as the case of A2af1a1 or A2r1, were not previously reported in native populations.

## Evaluation of population substructure on Santander department

### Genetic differentiation among provinces

To evaluate intrapopulation differentiation inside Santander department, samples were divided according to individual’s province of birth into: Comunera (*n* = 30), García Rovira (*n* = 30), Guanentá (*n* = 28), Metropolitana (*n* = 36), Soto Norte (*n* = 26), Vélez (*n* = 23), and Yariguíes (*n* = 31) (Figure 1B).

Diversity parameters of forensic relevance were re-calculated for each province and are detailed in Table 2. For the range 16024–576, differences on haplotype diversities among groups were <1%, while the exclusion power varied up to 5%. These differences were higher when discarding indels in homopolimeric tracts (Table 2). In these conditions, the haplotype diversity among departments varied up to 3% and the exclusion power up to 8%.

Population pairwise *F*_ST_ values were calculated using CR haplotypes both before and after discarding indels in homopolimeric tracts (Supplementary Table S5). The results obtained were similar in both analyses. Low *F*_ST_ values associated with high nondifferentiation *P-*values were obtained between some provinces, supporting a genetic homogeneity between them (Supplementary Table S5). Interestingly, the lowest *F*_ST_ was obtained between the populations from García Rovira and Vélez, two provinces that are geographically distant from each other. These populations are in mountainous areas of difficult access, remaining more isolated than other provinces. The isolation of these populations and the similar settlement, in the same period, can be behind their genetic similarity.

Although all *P*-values were above the significance level (0.0024, after Bonferroni correction), some comparisons showed meaningful genetic distances (3%–4%). This is the case of most pairwise comparisons including García Rovira and Vélez, the two most isolated provinces, and some pairwise comparisons including Soto Norte (Supplementary Table S5).

Although nondifferentiation *P*-values did not allow excluding sample size effects, the *F*_ST_ values observed in some pairwise comparisons can be due to differential native and/or continental contributions in each province. To test this hypothesis, a new analysis was performed after excluding the non-native haplotypes. The same levels of differentiation persisted (data not shown), showing that differences among provinces could not be solely explained by the proportion of African and European lineages, but also due to differences on the native backgrounds. This is clear by looking at the proportion of native haplogroups in the different provinces. For instance, haplogroup A reaches the highest frequency in García Rovira (53%) but is present at much lower proportion in Soto Norte (27%). This discrepancy in the native gene pool is also detected for macrohaplogroups B, C, and D, and may be behind some level of differentiation between provinces.

Because mtDNA is highly polymorphic, population differentiation based on mtDNA haplotype frequencies, calculated with *F*_ST_-based methods, may produce biassed estimates since unrealistic/very high sample sizes may be required to detect shared haplotypes. In the case of Santander, most haplotypes were only detected in a single province. To overcome this issue and try to clarify the results obtained, new *F*_ST_ analyses were performed using haplogroup instead of haplotype frequencies. Although the *F*_ST_ decreased, the differentiation pattern remained, with some high genetic distances associated with nonsignificant *P*-values (Supplementary Table S5). When samples were categorized by macrohaplogroup as A, B, C, D, R, or L, the same pattern emerged (data not shown), with genetic distances reaching 4.5%.

### Principal component analysis

**Figure 2:**
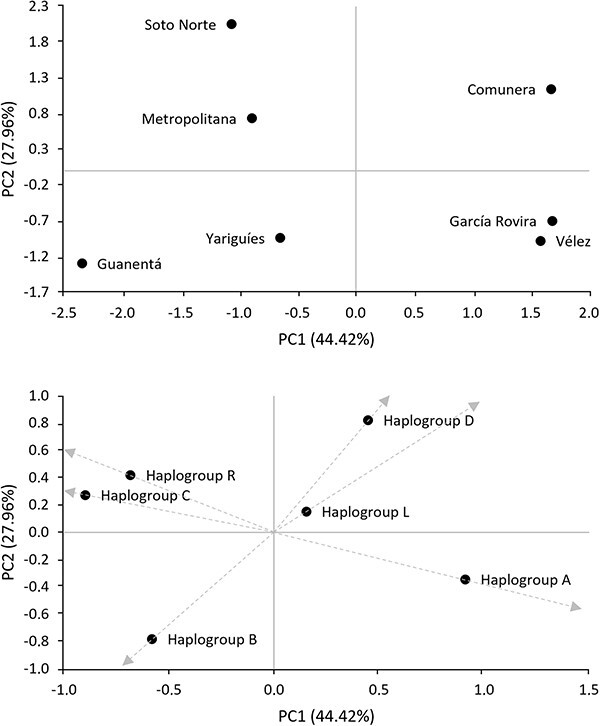
Principal component analysis (A) of mtDNA haplogroup frequencies (B) in Santander provinces.

A PCA based on macrohaplogroup frequencies (Figure 2) showed that native haplogroup frequencies contribute to the differentiation among populations. The first principal component separated García Rovira, Vélez, and Comunera from Soto Norte, Metropolitana, Yariguíes, and Guanentá, due to a higher frequency of haplogroups A/D and B/C, respectively. The second component separated Comunera from García Rovira and Vélez, and Yariguíes and Guanentá from Metropolitana and Soto Norte. García Rovira and Vélez presented a frequency of haplogroup A that is >50%; while Comunera, although having a high frequency of this haplogroup (43%), also had the highest input of haplogroup D. Guanentá and Yariguíes had ~40% frequency of haplogroup B, while Soto Norte and Metropolitana had the highest frequency of haplogroup C.

Apart from the native component, the European and African lineages also seemed to impact on provinces differentiation. The location of haplogroup R in the upper left quadrant of the PCA indicated that this lineage contributed to the separation of Metropolitana and Soto Norte from the remaining provinces. Although to a lesser extent, haplogroup L contributed to the separation of Comunera from the other populations.

Differences in the proportions of native and non-native ancestry between provinces were assessed through a Fisher test. Pairwise comparison between García Rovira and Soto Norte showed a significant *P*-value (*P* = 0.0071). García Rovira did not show non-native haplogroups while Soto Norte had the highest non-native input (23%).

### Analyses of molecular variance

Although high values of *F*_ST_ were found in some pairwise comparisons among provinces, nondifferentiation *P*-values were never significant. Nonetheless, according to the PCA, differences in haplogroup frequencies seemed to exist between some provinces.

Thus, AMOVA was performed considering different population groupings based on the previous results and different geographic and/or demographic criteria. In Cluster A, provinces were separated by the Eastern Andean range, into East (García Rovira, Comunera, Guanentá, Metropolitana, and Soto Norte) and West (Yariguíes and Vélez). In Cluster B, provinces were grouped considering the first principal component (representing 44.42% of the variation among provinces): Group 1 (Guanentá, Metropolitana, Soto Norte, and Yariguíes) and Group 2 (Comunera, García Rovira, and Vélez). In Cluster C, the two first principal components were taken into account, and samples were grouped according to the four quadrants of the PCA: Group 1 (García Rovira and Vélez), Group 2 (Guanentá and Yariguíes), Group 3 (Soto Norte and Metropolitana), and Group 4 (Comunera).

The AMOVA results showed no statistically significant variation among populations grouped into East and West of the mountain range, with a higher variation found within than among groups (Table 3). In contrast, clustering strategies based on PCA results allowed obtaining the highest values of among groups variation, associated to statistically significant nondifferentiation *P*-values (Table 3). Differences among groups were higher for Cluster B than Cluster C grouping strategy. These AMOVA results showed that grouping samples according to Cluster B would better reflect the genetic structure of the Santander population.

**Table 3:** AMOVA results for the three clusters tested.

Cluster	Among groups		Among populations within groups		Within populations	
	% of variation	Nondifferenciation probability	% of variation	Nondifferenciation probability	% of variation	Nondifferenciation probability
A	−0.76	0.6594 ± 0.0046	1.28	0.0905 ± 0.0029	99.48	0.1337 ± 0.0030
B	3.72	0.0280 ± 0.0017	−1.18	0.9477 ± 0.0022	97.47	0.1379 ± 0.0034
C	2.97	0.0090 ± 0.0009	−1.62	0.9780 ± 0.0015	98.66	0.1346 ± 0.0034

AMOVA: analysis of molecular variance.

## Conclusions

The analysis of the maternal lineages’ composition of Santander population showed a high preservation of the native ancestry, with just few haplogroups from African and European origin. These results are in line with the expected for Andean populations where European admixture was essentially mediated by men [6, 12]. In fact, a much higher proportion of autosomal (biparental) European ancestry (58%) was reported for the North East Andean populations (including those from Santander and Norte Santander departments) [5], supporting sex biassed intermarriages between European men and native women, typical from South American postcolonial populations [41, 49, 50].

The low contribution of African ancestry is explained by the low number of Africans that arrived in the department during the Atlantic slave trade. Historic records indicate that slaves arriving to Colombia would be taken mainly to the coast rather than to the interior of the country.

A high lineage diversity was found in Santander population, associated with a high MNPD among haplotypes. This heterogeneity is due to the coexistence of different native groups, and the arrival European settlers and African slaves during the colonial period. The multimodal mismatch distributions observed in all provinces indicate that, after an initial admixture process, the effective population size remained relatively stable, without major recent influxes that significantly changed the mtDNA background of the people living in the department.

When evaluating population substructure, most of the tests performed showed that differences between provinces are not statistically significant. However, both PCA and AMOVA results indicate that García Rovira and Veléz, the two most isolated provinces, can be differentiated from the remaining populations. A significantly higher proportion of native lineages were also found when comparing García Rovira with other provinces. Meanwhile, Soto Norte differed from the remaining provinces due to significantly higher proportions of non-native lineages. Although the differences described, the overall results point to the need of larger sample sizes to better elucidate the genetic structure of the Santander department.

Finally, since the maternal lineages background of Santander was still understudied, this work will support forensic investigations in Colombia by providing new data on mtDNA CR haplotype frequencies.

## Authors' contributions

Adriana Castillo, Leonor Gusmão and Fernando Rondón conceptualized the experiments and revised the manuscript; Adriana Castillo, Leonor Gusmão and Filipa Simão performed the experiments, analyzed the data, and drafted the manuscript; Adriana Castillo, Gerardo Mantilla, Leonor Gusmão and Filipa Simão participated in the experiments and data analyses; Adriana Castillo, Leonor Gusmão y Filipa Simão compiled the software. All authors contributed to the final manuscript and approved it.

## Funding

This work was supported by the Vice-rectorate of Investigation and Extension of the Industrial University of Santander [Project 2488 of 2019]. Filipa Simão and Leonor Gusmão were supported by Fundação de Amparo à Pesquisa do Estado do Rio de Janeiro – FAPERJ, Brazil [processes E-26/202.275/2019 and CNE-2022]. Leonor Gusmão is supported by Conselho Nacional de Desenvolvimento Científico e Tecnológico - CNPq, Brazil [ref.306342/2019-7].

## Compliance with ethical standards

Informed Consent, including the details of the study, was signed by the participants prior to donating the sample. This study was approved by the Ethics Committee of the Industrial University of Santander (21/05/21).

## Disclosure statement

None declared.

## Supplementary Material

Supplementary_Figures_owad032

Supplementary_Tables_R1_owad032
